# Yes-associated protein (YAP) induces a secretome phenotype and transcriptionally regulates plasminogen activator Inhibitor-1 (PAI-1) expression in hepatocarcinogenesis

**DOI:** 10.1186/s12964-020-00634-6

**Published:** 2020-10-23

**Authors:** Simone Marquard, Stefan Thomann, Sofia M. E. Weiler, Michaela Bissinger, Teresa Lutz, Carsten Sticht, Marcell Tóth, Carolina de la Torre, Norbert Gretz, Beate K. Straub, Jens Marquardt, Peter Schirmacher, Kai Breuhahn

**Affiliations:** 1 Institute of Pathology, University Hospital Heidelberg, Heidelberg, Germany; 2 Present address: Department of Medicine II, LMU Munich, Munich, Germany; 3 Medical Faculty Mannheim, Medical Research Center, University of Heidelberg, Mannheim, Germany; 4 Institute of Pathology, Johannes Gutenberg University, Mainz, Germany; 5 Department of Medicine I, Johannes Gutenberg University, Mainz, Germany; 6 Present address: Department of Medicine I, University Hospital Lübeck, Lübeck, Germany

**Keywords:** Hippo pathway, Liver cancer, Hepatocellular carcinoma, Transcriptional regulator, Oncogene

## Abstract

**Background:**

Overexpression and nuclear enrichment of the oncogene *yes-associated protein* (YAP) cause tumor initiation and support tumor progression in human *hepatocellular carcinoma* (HCC) via cell autonomous mechanisms. However, how YAP expression in tumor cells affects intercellular communication within the tumor microenvironment is not well understood.

**Methods:**

To investigate how tumor cell-derived YAP is changing the paracrine communication network between tumor cells and non-neoplastic cells in hepatocarcinogenesis, the expression and secretion of cytokines, growth factors and chemokines were analyzed in transgenic mice with liver-specific and inducible expression of constitutively active YAP (YAP^S127A^). Transcriptomic and proteomic analyses were performed using primary isolated hepatocytes and blood plasma. In vitro, *RNAinterference* (RNAi), expression profiling, functional analyses and *chromatin immunoprecipitation* (ChIP) analyses of YAP and the transcription factor *TEA domain transcription factor 4* (TEAD4) were performed using immortalized cell lines. Findings were confirmed in cohorts of HCC patients at the transcript and protein levels.

**Results:**

YAP overexpression induced the expression and secretion of many paracrine-acting factors with potential impact on tumorous or non-neoplastic cells (e.g. *plasminogen activator inhibitor-1* (PAI-1), *C-X-C motif chemokine ligand 13* (CXCL13), CXCL16). Expression analyses of human HCC patients showed an overexpression of PAI-1 in human HCC tissues and a correlation with poor overall survival as well as early cancer recurrence. PAI-1 statistically correlated with genes typically induced by YAP, such as *connective tissue growth factor* (CTGF) and *cysteine rich angiogenic inducer 61* (CYR61) or YAP-dependent gene signatures (CIN4/25). In vitro, YAP inhibition diminished the expression and secretion of PAI-1 in murine and human liver cancer cell lines. PAI-1 affected the expression of genes involved in cellular senescence and oncogene-induced senescence was confirmed in YAP^S127A^ transgenic mice. Silencing of TEAD4 as well as treatment with the YAP/TEAD interfering substance Verteporfin reduced PAI-1 expression. ChIP analyses confirmed the binding of YAP and TEAD4 to the gene promoter of PAI-1 (SERPINE1).

**Conclusions:**

These results demonstrate that the oncogene YAP changes the secretome response of hepatocytes and hepatocyte-derived tumor cells. In this context, the secreted protein PAI-1 is transcriptionally regulated by YAP in hepatocarcinogenesis. Perturbation of these YAP-dependent communication hubs including PAI-1 may represent a promising pharmacological approach in tumors with YAP overexpression.

**Video abstract**

**Supplementary information:**

**Supplementary information** accompanies this paper at 10.1186/s12964-020-00634-6.

## Plain English summary

Tumor cells communicate with other tumor cells and non-neoplastic cells to create a growth-supportive environment. Indeed, a comprehensive understanding of these communication networks allows to investigate novel therapeutic strategies that ‘disturb’ abnormal crosstalk between these cells. The goal is to normalize the tumor environment and to counteract tumor growth. In this study, we focus on the oncogene YAP (*yes-associated protein*) that causes liver tumor formation. Using independent and complementary screening techniques and model systems, we identified several secreted factors that are induced by YAP in liver cells (secretome phenotype) and that could adjust cell-cell communication in a tumor-supporting manner. For example, high YAP-dependent *plasminogen activator inhibitor-1* (PAI-1) levels in liver cancer patients associate with poor clinical outcome. PAI-1 regulates the expression of factors associated with cellular senescence. Mechanistically, we show that YAP together with a transcription factor of the *TEA domain transcription factor* family TEAD4 controls PAI-1 expression and secretion. We conclude that oncogenes such as YAP control the expression of secreted factors to generate a tumor-supportive microenvironment. This paracrine communication could serve as adjusting screw for the normalization of cell communication.

## Background

The Hippo signaling pathway and its negatively regulated downstream effector *yes-associated protein* (YAP) control tissue growth and organ size in embryogenesis as well as under regenerative conditions [1]. As described for the liver in great detail, dysregulation of the pathway is associated with nuclear YAP enrichment, which is leading to uncontrolled hepatocellular proliferation and malignant transformation [2]. YAP overexpression in about 30% of all cases defines liver cancer patients (*hepatocellular carcinoma*; HCC) with shorter survival and early cancer recurrence, illustrating the necessity to understand how this protein supports tumorigenesis [3].

So far, different YAP-driven mechanisms that contribute to tumor cell initiation and progression have been described. For the liver, they include the expansion of cells with a progenitor phenotype, which are the origin for the development of liver tumors with mixed differentiation [4–6]. YAP also supports migration/invasion in a tumor cell autonomous manner [7]. Lastly, YAP-mediated hepatocellular proliferation leads to *chromosome instability* (CIN) and accumulation of genetic alterations, which represent the basis for tumor initiation [3, 8]. Next to these tumor cell-autonomous mechanisms, heterologous cell communication via secreted proteins could contribute to carcinogenesis. Indeed, recent data illustrate that YAP overexpression in tumor cells controls the expression and secretion of growth factors and cytokines already in early stages of hepatocarcinogenesis, creating a tumor-supporting microenvironment [9]. Interestingly, YAP-dependent paracrine-acting proteins produced by hepatocytes and tumor cells may affect non-parenchymal liver cells (e.g. endothelial cells, hepatic stellate cells, and Kupffer cells) or they may control the behavior of hepatocellular cells in an autocrine manner. However, if the Hippo/YAP signaling axis directly controls these paracrine-acting factors via transcriptional regulation or if expression of these proteins is the consequence of secondary effects is not well understood. In addition, the relevant downstream effectors that mediate biologically relevant processes of YAP in carcinogenesis are not well-defined. Lastly, identification of YAP-dependent communication hubs could lead to the development of specific therapies. Targeting these potential ‘points of interference’ would be of special interest for patients with YAP overexpression.

To understand how YAP expression in HCC cells could affect the production of secreted factors, different technical approaches were used. By using transcriptome and proteome analyses, we describe the secretome of primary murine hepatocytes that overexpress active YAP. As exemplified for *plasminogen activator inhibitor-1* (PAI-1), we show that overexpression of this factor in human *hepatocellular carcinoma* (HCC) patients correlates with poor clinical outcome and expression of known YAP target genes. In addition, PAI-1 controls the expression of genes involved in cellular senescence. Together with *TEA domain transcription factor 4* (TEAD4), YAP transcriptionally regulates PAI-1 expression in human and murine liver cancer cell lines.

## Materials and methods

Sequences for siRNAs and primers (mouse and human) as well as antibodies (incl. dilutions and applications) are listed in Supplementary Tables S1, S2, S3, S4.

### Mouse work

LAP-tTA/Col1A1-YAP^S127A^ (LAP-YAP) mice were used in this study [6, 10]. For transgene repression of constitutively active YAP^S127A^, mice received 2 mg/ml doxycycline in their drinking water supplemented with 10 mg/ml sucrose. For transgene induction, doxycycline was withdrawn at the age of 10 weeks. Control mice (with doxycycline) and animals with YAP^S127A^ expression were sacrificed at time points that are indicated below.

### Isolation of primary hepatocytes and extraction of plasma samples

Primary murine hepatocytes were isolated from LAP-tTA/Col1A1-YAP^S127A^ (LAP-YAP) mice 12 weeks after doxycycline withdrawal. Cells were cultivated on collagen I-coated 10 cm dishes (Corning Life Sciences, Amsterdam, Netherlands) in Williams adhesion medium (Biochrom, Berlin, Germany) containing 100 nM dexamethasone, 2 mM glutamin, 10% FCS and 1% penicillin/streptomycin (Sigma-Aldrich, Taufkirchen, Germany) for 4 h at 37 °C to allow attachment. After removing non-adherent cells, attached hepatocytes were cultured for 24 h and subjected to protein and RNA isolation. No obvious changes between wildtype and YAP^S127A^ transgenic hepatocytes with regard to cell adhesion were observed.

For the collection of murine blood plasma (13 weeks after YAP^S127A^ induction), mice were euthanized by an intraperitoneal injection using Ketamin/Xylazine and whole blood was obtained through an intracardial puncture using a heparin-coated 1 ml syringe (BD, Heidelberg, Germany). To obtain blood plasma, samples were centrifuged (30 min, 2000 x *g* at room temperature). Plasma samples were stored at − 80 °C.

### Cell culture, RNA-interference, and Verteporfin treatment

The murine HCC cell line Hepa1–6 (CLS, Eppelheim, Germany) as well as the human cell lines Sk-Hep1 (ATCC; LGC Standards, Wesel, Germany), SNU-182 (ATCC; LGC Standards), HEK-293 cells were cultured in DMEM and RPMI, (Sigma-Aldrich) supplemented with 10% FCS (Gibco/Life Technologies) and 1% penicillin/streptomycin (Sigma-Aldrich) at 37 °C and 5% CO_2_, respectively. Cells were seeded in 6-well plates 1 day prior to transfection with gene-specific small interfering RNAs (siRNAs). siRNAs were received from Eurofins MWG Operon (Ebersberg, Germany) and diluted in OptiMEM (Gibco/Life Technologies) to a final concentration of 20 or 40 nM. Oligofectamine (Life Technologies) was used as transfection reagent according to the manufacturer’s protocol. After incubation at room temperature for 15 min, the reagents were mixed, incubated for another 10 min at room temperature and distributed onto the cells covered with OptiMEM. One millilitre DMEM was added after 4 h. The medium was replaced by FCS-free medium after 24 h. Cells and supernatant were harvested 48 h after transfection. Untreated cells and scrambled siRNA-transfected cells (scr.) were used as controls as indicated.

The YAP/TEAD-Inhibitor Verteporfin (Sigma-Aldrich) was dissolved in DMSO to prepare a stock solution of 2 mM according to the manufacturer’s instructions. One day after seeding, cells were treated with Verteporfin (0.25 to 1 μM). Medium was changed after 24 h and cells/supernatant were harvested after 48 h. DMSO-treated cells served as control (max. volume fraction of 0.05% DMSO in cell culture medium).

### Preparation of total RNA, reverse transcription and real-time PCR

For isolation of total RNA from cultured cells, the NucleoSpin RNA II kit (Machery-Nagel, Düren, Germany) was applied. For RNA extraction from tissue samples, the Precellys Ceramic Kit 1.4 and the Precellys 24 Homogeniser were used (Peqlab, Erlangen, Germany), followed by RNA purification using the NucleoSpin RNA II kit.

For cDNA-synthesis, 1 μg of total RNA was reverse transcribed using the Revert Aid H Minus RT kit (Thermo Fisher Scientific, Darmstadt, Germany). Semi-quantitative real-time PCR was performed with ABsolute qPCR SYBR Green ROX Mix (Steinbrenner, Wiesenbach, Germany) using the following cycling program: 95 °C for 15 min followed by 40 cycles of 95 °C for 15 s and 60 °C for 1 min. Melting curve analysis was applied for the validation of product specificity. For human cells, β2-microglobulin (B2M) and ribosomal protein L41 (RPL41) were used for normalization. For experiments with murine cells, β-actin (ACTB) was used as housekeeping gene.

For tissue analyses, stable genes for normalization were identified by the GeNorm software [11]. The panel of reference genes for human tissue samples included B2M, peptidylprolyl isomerase A (PPIA), RPL41, TATA-box binding protein (TBP) and serine and arginine rich slicing factor 4 (SRSF4). For mouse samples, ACTB, glyceralaldehyd-3-phosphate dehydrogenase (GAPDH), hypoxanthine-phosphoribosyltransferase (HPRT) and PPIA were measured.

### Protein isolation, acetone precipitation and Western immunoblotting

For isolation of total protein from cultured cells, 10x Cell Lysis Buffer (Cell Signaling/New England Biolabs, Frankfurt, Germany) supplemented with PhosStop (Roche, Mannheim, Germany) and Protease-Inhibitor Mix G (SERVA, Heidelberg, Germany) were used. Protein isolation from tissue samples (13 weeks after YAP^S127A^ induction) was performed using the Precellys Ceramic Kit 1.4 and the Precellys 24 Homogeniser (Peqlab). Supernatant containing protein lysates was collected after sonication and centrifugation.

For detection of secreted proteins in cell culture supernatant, acetone precipitation was performed. After cell debris was removed by centrifugation, 1.5 ml of the samples were mixed with 6 ml ice-cold acetone and incubated at − 20 °C for 1 h. Samples were centrifuged at 3800 x *g* at 4 °C for 30 min to form a protein pellet. After discarding the supernatant, the pellet was dried for 30 min at room temperature and resuspended in 300 μl lysis buffer.

Protein amounts were measured with the NanoDrop ND-1000 spectrophotometer (Thermo Fisher Scientific; wavelength: 280 nm). After dilution in loading buffer (2% SDS, 10% glycerol, 5% β-mercaptoethanole, 0.002% bromphenol blue, 62.5 mM Tris-HCl), equal amounts of total protein per lane were separated by 8–12% sodium dodecyl sulfate (SDS) polyacrylamide gel electrophoresis and blotted on a nitrocellulose membrane (GE Healthcare, Solingen, Germany). The membranes were blocked with 5% milk or bovine serum albumin (BSA) in TBS-T (Tris-buffered saline/Tween 20). All primary antibodies were added and incubated at 4 °C overnight. All secondary antibodies were also diluted in a 5% milk and BSA/TBS-T solution, respectively (1:20,000; IRDye 680 and 800, LI-COR Biosciences, Bad Homburg, Germany) and incubated at room temperature for 1 h. Images were acquired with the Odyssey Sa Infrared Imaging System (LI-COR Biosciences). For normalization, the appropriate housekeeping gene was detected (ACTB and GAPDH for cell culture samples, β-tubulin for tissue samples, and albumin for plasma samples).

### Chromatin-Immunoprecipitation (ChIP)

Using the JASPAR database, the promoter of human and mouse SERPINE1 gene (coding for PAI-1) was searched for potential binding sites of TEAD4 [12]. Negative control primers were designed based on random sequences downstream of the potential TEAD4 binding sites. The ChIP-analysis was performed as previously described [3]. Promoter binding was compared to an input sample, which was removed and frozen at − 20 °C prior to sample preclearing. Crosslink reversion and purification of DNA of the input sample were performed as for the ChIP samples.

### Luciferase assay

Assay was performed as previously described [3]. In brief, a 250 fragment of the human *SERPINE1* promoter was cloned in the pGL3 basic firefly luciferase vector (pGL3-PAI-1) using MluI/XhoI restriction enzymes. pGL3-PAI-1 was transiently transfected with the pRL-CMV-Renilla vector (ratio: 1:1 or 1:4). For inhibition experiments, cells were first transfected with the respective siRNAs for 24 h, followed by transfection with the pGL3/pRL vectors. The mutation in the TEAD4 binding site was included using the following primer for: 5′-CAG CAG CTG AA**C** TCC TGC AGC TCA G-3′ and rev: 5′-CTG AGC TGC AGG A**G**T TCA GCT GCT G-3′. Underlined: TEAD4 binding site; bold: mutated base. Amplification of fragments was done using the Phusion polymerase (Thermo Fisher Scientific). For YAP overexpression, human YAP was cloned into a pDEST vector.

### Expression profiling

For the analysis of PAI-1 dependent gene expression, PAI-1 was transiently silenced in human Sk-Hep1 cells using siRNA. Cells were harvested 2 days after transfection and total RNA was isolated. RNAi efficiency was validated by real-time PCR and western immunoblotting.

For expression profiling of primary isolated hepatocytes and immortalized cells after PAI-1 inhibition, only samples with a *RNA integrity number* (RIN) > 7 were used. Purified and fragmented complementary DNA was generated according to the manufacturer’s instructions (Thermo Fisher Scientific, Waltham, MA, USA). Fragments were biotin-labelled prior to hybridization on MoGene-2_0-st chips using a GeneChip Hybridisation oven 640. Successive staining and scanning were performed with a GeneChipFluidics Station 450 and a GeneChip Scanner 3000, respectively (Thermo Fisher Scientific).

After gene annotation, the fluorescence intensity was measured, normalized, and differential expression was statistically assessed using the software package SAS JMP7 genomics (SAS Institute, Cary, NC, USA). As cut-off, a *false discovery rate* (FDR) value of 0.05 was considered as significant. To assure a homogeneous distribution of the generated data, *principal component analysis* (PCA) was performed to compare the similarity of individual biological samples in this study. To identify pathways and cellular processes with significant enrichment of differentially expressed genes, gene set enrichment analysis (GSEA) was performed. Identified KEGG pathways, including “Cytokine – Cytokine Receptor Interaction – mmu04060”, were used for heatmap generation. For the transcriptomic response after transient inhibition of PAI-1, the KEGG pathway “cellular senescence – hsa04218” and its normalized enrichment score were used.

### Antibody array

For proteome analysis of murine blood plasma samples, the Mouse XL Cytokine Array Kit by R&D Systems (Minneapolis, USA) was applied according to the manufacturer’s protocol. Plasma samples of wildtype (*n* = 3) and YAP^S127A^ mice (*n* = 5) were analyzed. After incubating with biotinylated detection antibodies for 1 h at room temperature, diluted IRDye 800CW streptavidin was administered and the arrays were scanned using Odyssey Sa Infrared Imaging System (LI-COR Biosciences). Background signal was subtracted, and average intensity of the reference spots was used for normalization.

### Immunohistochemistry

For immunohistochemical staining, formalin-fixed and paraffin embedded tissue sections were first deparaffinized and rehydrated by washing with xylene (3x for 6 min each time), 100% ethanol, 96% ethanol, 70% ethanol (each 2x for 5 min each time) and finally rinsed in distilled H_2_O. Antigen retrieval was performed in a pressure cooker for 15 min by covering the slides with Target Retrieval Solution (citrate buffer, pH 6.0; DAKO, Hamburg, Germany). After cooling down the slides for 30 min and washing with TBS (2x for 5 min each time), the primary antibody was applied for 1 h. Slides were washed again with TBS twice for 5 min prior to incubation with the biotinylated secondary antibody for 25 min at room temperature, followed by 2 × 5 min each time TBS washes, 10 min H_2_O_2_ block, 2 × 5 min each time TBS washes and Streptavidin-HRP (DAKO) incubation for 25 min. After 2 × 5 min each time TBS washes, peroxidase was detected by AEC (3-amino-9-ethylcarbazole, DAKO) followed by a haematoxylin stain.

### β-Galactosidase staining

For the detection of senescent cells, β-galactosidase stain was performed using frozen sections of murine liver tissue from wildtype (*n* = 8) and YAP^S127A^ mice (*n* = 8). After fixation in 0.5% glutaraldehyde/PBS for 15 min, slides were washed once with PBS and twice with PBS (pH 5.5) supplemented with 1 mM MgCl_2_ (each for 5 min). Freshly prepared X-Gal staining solution (92.5% PBS/MgCl2, 5% 20x KC-Buffer, 2.5% 40x X-Gal (Roche)) was applied and incubated overnight at 37 °C in a wet chamber, followed by 3 × 5 min PBS washes and counterstaining with eosin. For quantification of β-galactosidase positive cells, the FIJI (https://fiji.sc/) segmentation tool “Trainable WEKA Segmentation” was used.

### Human samples

Transcriptomic and survival data of a human HCC cohort from 242 patients were reanalyzed [13]. Independent RNA samples from 20 HCC patients were surgically resected at the University Hospital of Mainz.

### Tissue microarray

The tissue microarray (TMA) contained 7 non-tumorous liver tissues and 91 HCC tissues (grading: 7x G1, 66x G2, 14x G3, 4x G4). The evaluation of the tissue samples was performed by a scoring system depending on the percentage of positive tumor cells (0 = no expression, 1 = less than 1% positive, 2 = 1–9% positive, 3 = 10–50% and 4 = more than 50% positive cells) and staining intensity (0 = negative, 1 = low, 2 = medium, 3 = strong) of the immunohistochemical staining. The product of both values resulted in a score ranging from 0 to 12. A score equal to or higher than 6 was defined as overexpression of the investigated protein. The analysis was performed by two experienced investigators (S.M., M.T.).

### Data analysis and statistics

Western immunoblotting, ChIP analysis and qPCR experiments were biologically repeated 2 or 3 times. For statistical analysis and graph preparation the software Excel and SPSS were used. Data are shown as mean ± standard deviation. For statistical comparison of two independent groups, the Mann-Whitney U test was applied. Survival and recurrence data of cancer patients were analyzed using the with log-rank test. Spearman’s rho was used to determine statistical dependence of ordinal scaled data. Significance levels were defined as p* ≤ 0.05, p** ≤ 0.01 and p*** ≤ 0.001. Heatmaps were created using the online tool Morpheus (https://software.broadinstitute.org/morpheus/).

## Results

### YAP regulates the hepatocellular secretome in an HCC tumor model

In order to obtain a comprehensive overview of how YAP may affect the tumor microenvironment in a paracrine manner, a mouse model with inducible expression of human YAP lacking the phosphorylation site at amino acid 127 (YAP^S127A^) was used [3, 10]. For this, mice carrying a doxycycline-dependent allele for YAP^S127A^ were crossed with mice expressing the tetracycline transactivator (tTA) under control of the liver activator protein (LAP) promoter (Tet-off system). Withdrawal of doxycycline in these mice induced a robust overexpression of constitutively active YAP^S127A^ compared to control animals. This YAP^S127A^ expression caused severe hepatomegaly after 8–12 weeks and tumor formation after 12–15 weeks of transgene induction [3, 6].

**Fig. 1:**
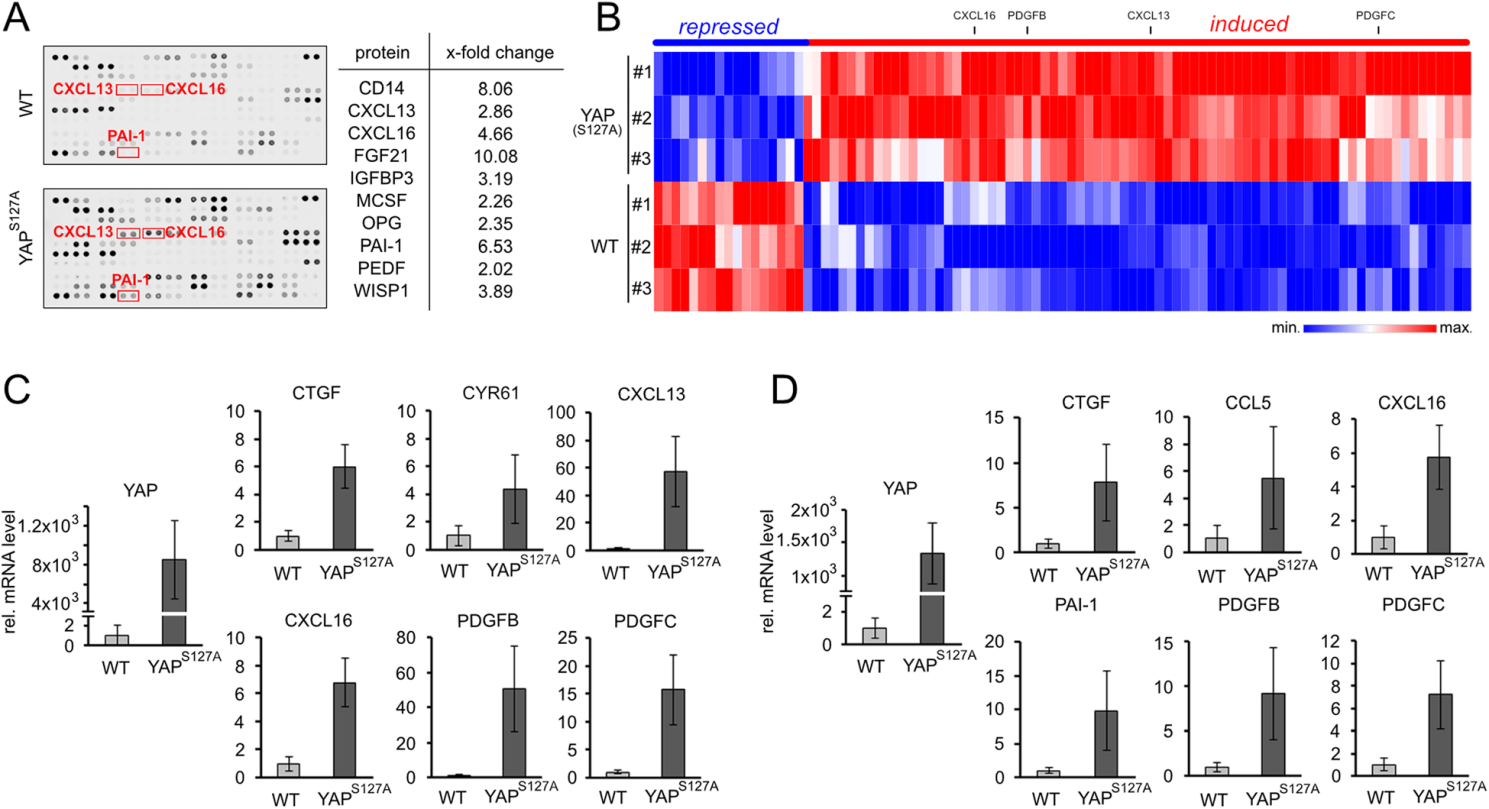
Identification of the YAP-dependent secretome in hepatocytes. **a** Exemplary proteome profiling of blood plasma isolated from WT and YAP^S127A^-transgenic mice revealed significantly different expression of 24 cytokines (17 up and 7 downregulated), (*n* = 3–5/group). Table summarizes 10 significantly elevated factors that show at least 2-fold induction in plasma samples from YAP^S127A^-transgenic mice. **b** Heatmap illustrating expression of significantly regulated genes annotated in the KEGG pathway ‘cytokine-cytokine receptor signaling’ (KEGG: mmu04060) in primary hepatocytes isolated from WT and YAP^S127A^-transgenic mice 12 weeks after doxycycline withdrawal (*n* = 3/group). Genes selected for further validation are indicated. **c** Confirmatory real-time PCR of genes selected from (**b**) was performed on independent samples derived from primary WT and YAP^S127A^-positive hepatocytes, (*n* = 5–7/group). **d** Real-time PCR analysis of selected candidates on samples isolated from whole tissue lysates of WT and YAP^S127A^-transgenic mice, (*n* = 10–16/group)

In a first step, cytokine abundance in blood plasma samples derived from wildtype (WT) and YAP^S127A^ transgenic mice was analyzed using a proteome profiler array (*n* = 111 cytokines). The quantitative analysis revealed that 10 proteins were significantly induced at least 2-fold in blood samples from YAP^S127A^ mice (Fig. 1a). These factors included *C-X-C motif chemokine ligand 13* (CXCL13), CXCL16 and *plasminogen activator inhibitor-1* (PAI-1; synonym: *serine protease inhibitor E1* (SERPINE1)).

However, this screen was not informative regarding the cellular source of the secreted proteins. For this reason, we decided to further examine primary hepatocytes isolated from WT and YAP^S127A^ expressing mice using transcriptome analysis (*n* = 3 from each group). Expression profiling revealed that the expression of 2212 genes differed significantly between WT and YAP^S127A^ hepatocytes. Interestingly, many of these differentially expressed genes were secreted factors or were involved in signal transduction as exemplified for cytokine-cytokine receptor interaction (KEGG pathway mmu04060), (Fig. 1b). For this pathway, 93 genes were differentially expressed (FDR ≤ 0.05), with many secreted factors and receptors induced in YAP^S127A^ hepatocytes indicating that YAP induced a secretory response (Fig. 1b, supplementary Table S5). In total, 47 of the significantly regulated factors were receptors, while 46 genes represented secreted ligands. For further comparative analyses, we focused on ligands that were positively regulated in YAP^S127A^-transgenic hepatocytes (38/47).

For confirmation, YAP^S127A^-dependent expression of known target genes such as *connective tissue growth factor* (CTGF) and *cysteine rich angiogenic inducer 61* (CYR61) were analyzed by real-time PCR using samples derived from primary isolated murine hepatocytes (Fig. 1c). In addition, overexpression of several identified paracrine-acting factors in YAP^S127A^-positive cells was demonstrated including CXCL13, CXCL16, *platelet derived growth factor* subunit B (PDGFB) and PDGFC (Fig. 1c). Lastly, factors that were identified in blood plasma samples at protein level and in primary hepatocytes at transcript level were analyzed in tissue lysates derived from WT and YAP^S127A^ transgenic mice (Fig. 1d). Elevated expression of these candidates was also confirmed in tissue lysates derived from YAP^S127A^ expressing mice (e.g. PAI-1, CXCL16).

**Fig. 2:**
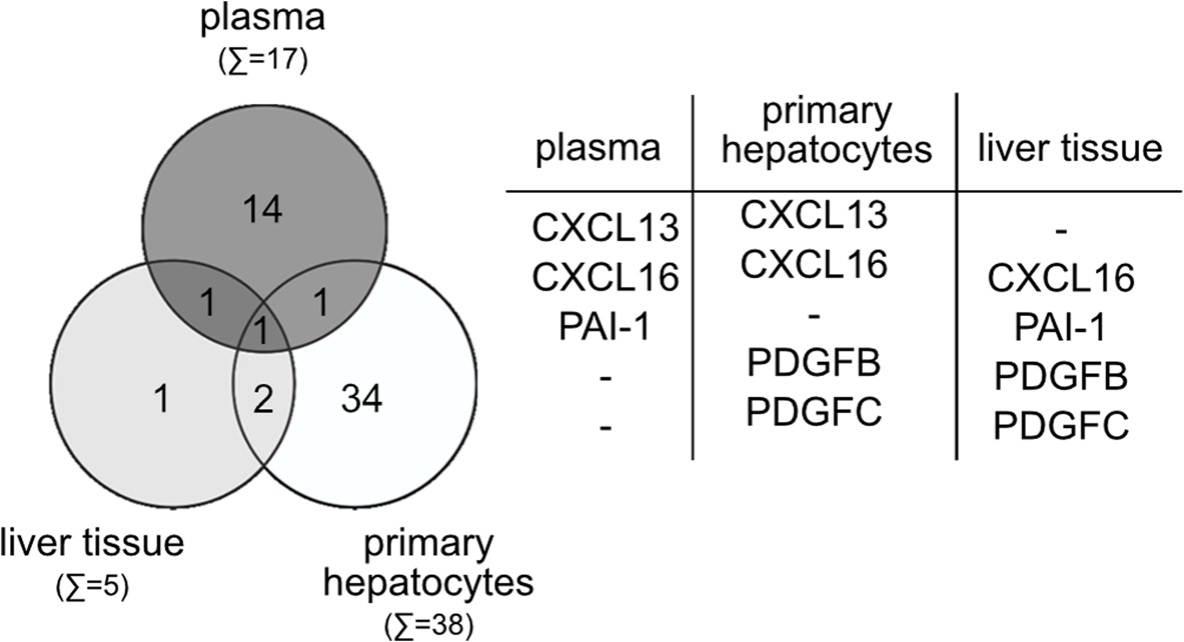
Identification of PAI-1 as potential YAP target gene in HCC cells. Intersection analysis comparing the different technical approaches revealed that 5 secreted candidates were identified by at least 2 experiments (listed in table)

In the last step, a comparative analysis of protein (plasma) and transcript data (primary hepatocytes as well as liver tissues) was performed. In total, 5 potential YAP target genes were regulated in at least two of the performed approaches (Fig. 2).

Together, these data illustrate that in vivo YAP controls the expression of several secreted factors, which may individually contribute to the modulation of the microenvironment and tumor cells in a paracrine manner.

### Overexpression of YAP-regulated PAI-1 correlates with unfavorable HCC prognosis

**Fig. 3:**
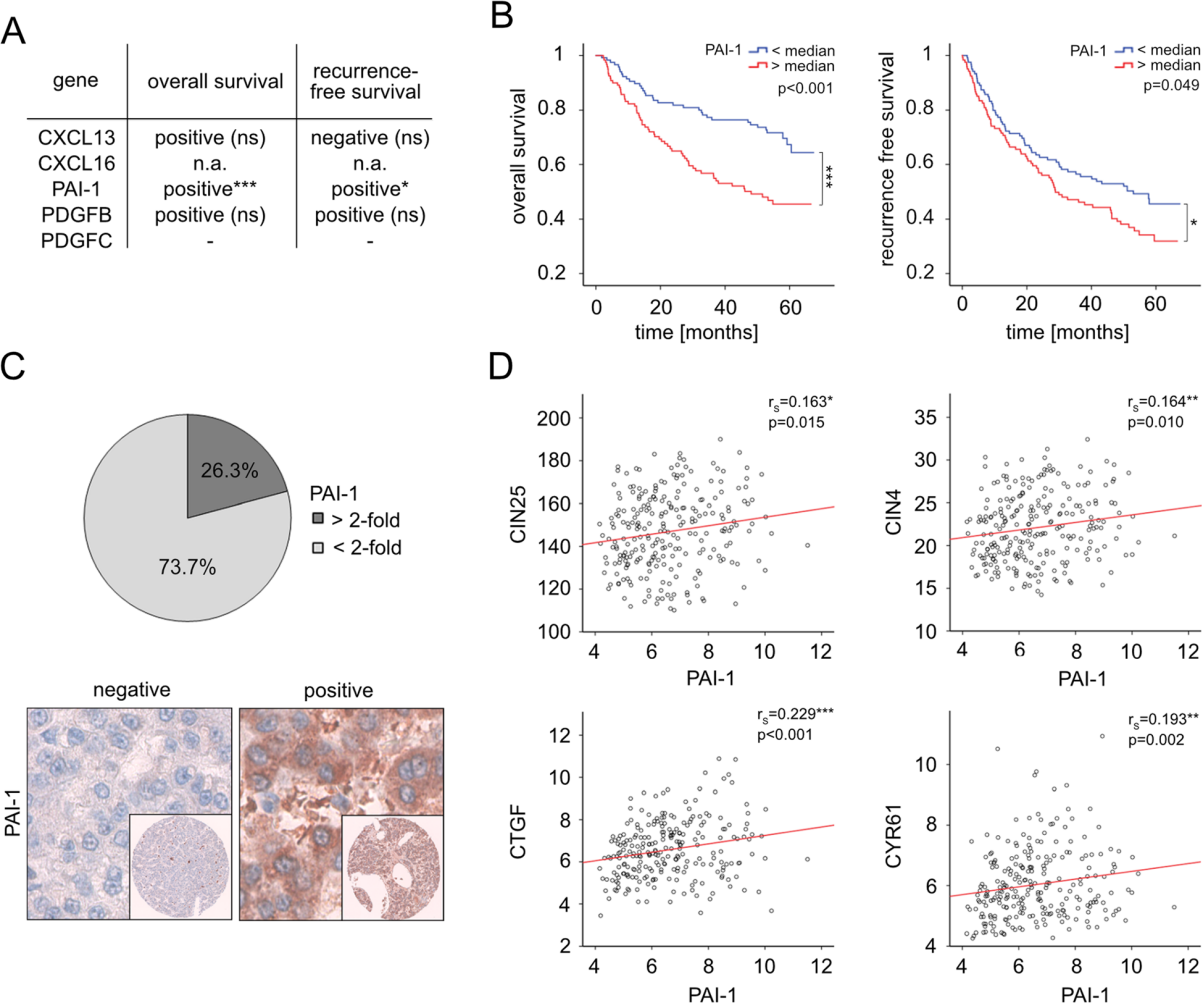
PAI-1 expression in HCC tissues associates with poor clinical outcome and YAP target gene expression. **a** Table summarizing the results of patient sample analysis. Positive: high level expression positively associates with poor clinical outcome; negative: high level expression negatively associates with poor clinical outcome. Transcriptome data were derived from 242 HCC patients. ns - no significant difference, *p ≤ 0.05, ***p ≤ 0.001. Statistical test: log-rank test. **b** Overall survival and cancer recurrence of HCC patients with high or low PAI-1 transcript levels are shown by Kaplan-Meier curves. *p ≤ 0.05, ***p ≤ 0.001. Statistical test: log-rank test. **c** Pie chart illustrating ≥2-fold PAI-1 overexpression at the mRNA level in HCC patient samples compared to corresponding normal tissue (real-time PCR). In the lower part, exemplary immunohistochemical stains of HCC tissues with low or high PAI-1 expression are shown (TMA analysis). **d** Association between PAI-1 expression and YAP-induced gene signatures (CIN4/CIN25) as well as YAP target genes CTGF and CYR61 in human HCC tissues is shown. *p ≤ 0.05, **p ≤ 0.01, ***p ≤ 0.001. Statistical test: Spearman correlation (r_s_)

We then decided to investigate if the secreted factors CXCL13, PAI-1, PDGFB and PDGFC also correlated with HCC patient prognosis and analyzed a cohort consisting of 242 HCC cases [13], (data for CXCL16 was not available). For this, we correlated the abundance of the remaining 4 candidates with survival and tumor recurrence. Interestingly, only for PAI-1 a robust and significant association between high-level expression and poor clinical outcome was detectable (Fig. 3a/b). For this reason, all further analyses focused on the role of PAI-1 in hepatocarcinogenesis.

First, PAI-1 overexpression was substantiated in an independent HCC cohort consisting of 19 HCC tissues and corresponding non-tumorous liver tissues. Indeed, real-time PCR analysis revealed an at least 2-fold induction of PAI-1 in about 26% of all examined HCC tissues compared to the control samples (Fig. 3c, upper panel). Elevated PAI-1 and YAP protein levels (IHC score ≥ 6) could be detected in 25 and 24% of HCC patients by analyzing PAI-1 and YAP stainings (Fig. 3c, lower panel). However, a significant correlation between nuclear YAP positivity and PAI-1 was not detectable in this cohort.

YAP transcript levels were in most cases not or only moderately altered in HCC cells, since its aberrant activation is caused by nuclear translocation. For this reason, we tested if PAI-1 expression was associated with the expression of typical YAP target genes and YAP-induced gene signatures, which can be considered as an approximation for YAP activity [3]. Indeed, a moderate but significant association between PAI-1, CTGF and CYR61, as well as the YAP-dependent CIN signatures (CIN25 and CIN4) was observed in samples derived from HCC patients (Fig. 3d). This statistical analysis illustrated that transcriptional YAP activity in HCC tissue correlated with increased PAI-1 expression.

In sum, the overexpression of the potential YAP target gene PAI-1 was associated with worse clinical outcome of HCC patients and its expression correlated with typical YAP downstream targets genes.

### YAP regulates PAI-1 expression and secretion

In our initial screening approaches, differences at transcript (in tissue samples) or protein levels (in blood samples) could be caused by cells that were negative for YAP (e.g. non-parenchymal cells). For this reason, our findings of YAP-dependent PAI-1 expression were confirmed using immortalized cancer cell lines of hepatocellular origin.

**Fig. 4:**
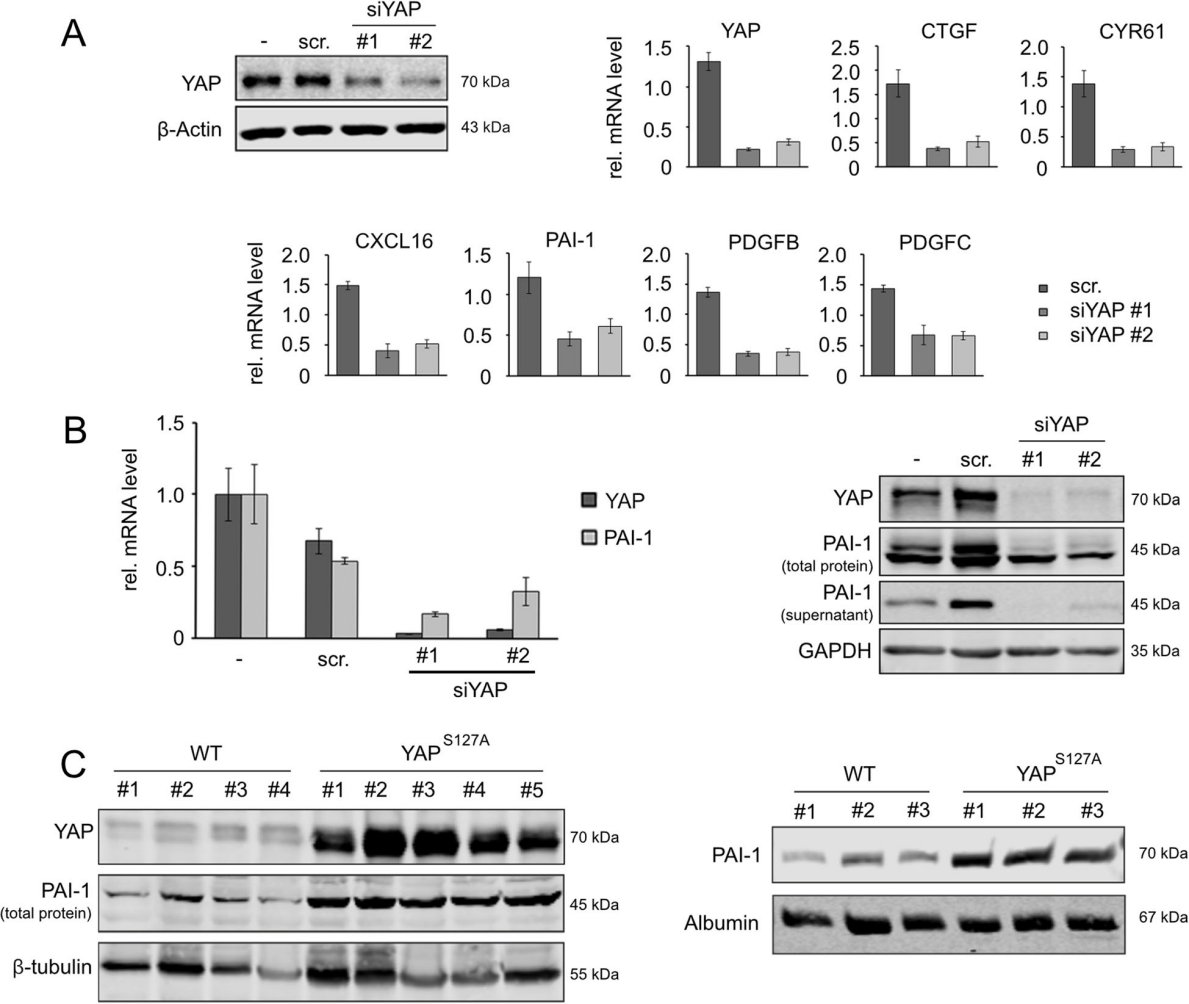
YAP transcriptionally regulates PAI-1 expression and extracellular PAI-1 levels. **a** SiRNA-mediated YAP inhibition in murine Hepa1–6 cells using two independent siRNAs (#1, #2). Analysis was performed 48 h after transfection. Western immunoblot and real-time PCR confirmed efficient YAP silencing. Additional real-time PCR analysis was performed for secreted factors identified after initial expression profiling. **b** Real-time PCR for YAP and PAI-1 after siRNA-dependent YAP inhibition (Sk-Hep1, left panel). Western immunoblot of total PAI-1 amounts and secreted PAI-1 after YAP inhibition (right panel). The scrambled siRNA transfected control was used for normalization and to calculate relative changes. **c** Western immunoblot of total YAP and PAI-1 levels in total protein extracts derived from murine WT and YAP^S127A^ transgenic livers followed by densitometric quantification (WT: 1 ± 0.26; YAP^S127A^: 4.41 ± 1.03, left panel). Detection of secreted PAI-1 in blood plasma samples derived from WT and YAP^S127A^ animals followed by signal quantification (WT: 1 ± 0.16; YAP^S127A^: 2.95 ± 0.19, right panel). Western immunoblot signals for panel **b** and **c** were quantified and normalized to the respective house-keeping genes. ‘scr’ controls represent samples that were transfected with the transfection reagent and non-specific siRNA (40 nM for murine Hepa1–6 cells and 20 nM for human Sk-Hep1 cells) with no similarity to cDNA

First, silencing of YAP by *RNAinterference* (RNAi) was performed in the murine HCC cell line Hepa1–6 using two independent siRNAs. In comparison to untreated and scrambled siRNA-transfected cells, the efficient knockdown of YAP led to reduced transcript levels of several identified factors including CTGF, CYR61, CXCL16, PDGFB/C and PAI-1 (Fig. 4a). Second, we specifically tested the impact of YAP silencing on PAI-1 expression in a human cancer cell line. As expected from the previous results, YAP silencing reduced PAI-1 transcript abundance (Fig. 4b, left panel). More important, the amount of PAI-1 in total protein extracts and cell culture supernatant was diminished by 50 and 90%, respectively, further supporting the idea that PAI-1 might mediate YAP-induced cell extrinsic effects (Fig. 4b, right panel). Lastly, significantly elevated PAI-1 protein levels were detectable in total protein extracts of liver tissues as well as plasma samples derived from mice with inducible YAP^S127A^ expression (Fig. 4c).

Together, these results from murine and human model systems show the YAP-dependent regulation of PAI-1 expression.

### YAP-induced PAI-1 controls expression of genes involved in cellular senescence

**Fig. 5:**
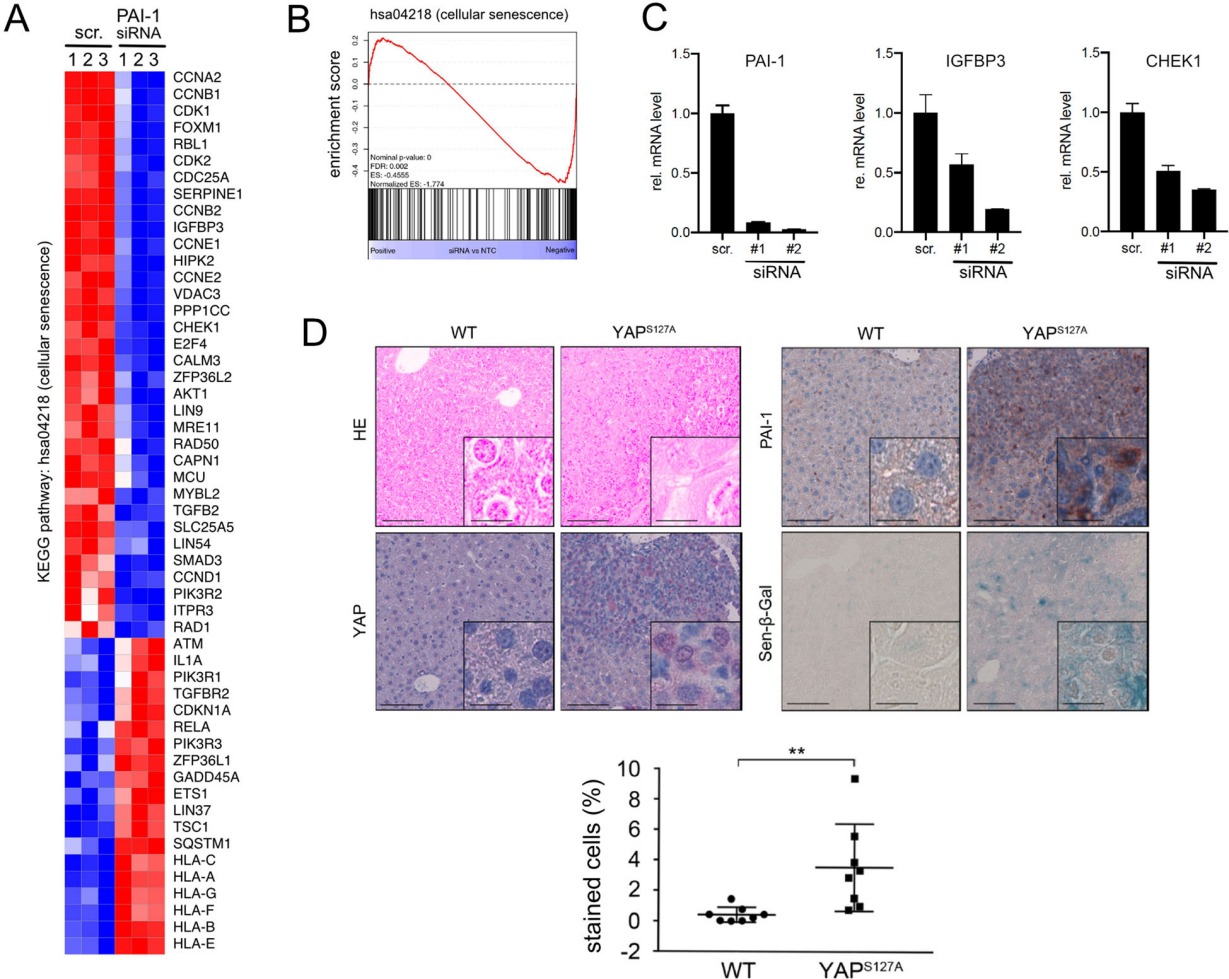
Functional relevance of PAI-1 in liver cancer. **a** Expression profiling was performed after siRNA-mediated inhibition of PAI-1 for 48 h. Scrambled siRNA-transfected cells served as controls (scr.). A heatmap consisting of 54 differentially regulated mRNAs of the KEGG pathway ‘cellular senescence’ is shown (hsa04218). Depicted genes are significantly regulated by at least +/− 1.3-fold (FDR ≤ 0.05). **c** GSEA graph for the KEGG pathway ‘cellular senescence’ illustrating the enrichment of genes after PAI-1 silencing. Scr. - transfection of scrambled siRNA. **d** Confirmatory real-time qPCR using two independent siRNAs targeting PAI-1. PAI-1 and the senescence-associated genes IGFBP3 and CHEK1 were measured. **e** H/E, immunohistochemical and β-galactosidase stains of tissue samples derived from WT and YAP^S127A^-transgenic mice (scale bar: lower magnification, 100 μm; higher magnification, 20 μm). Boxplot diagram illustrates significantly increased β-galactosidase staining in livers from YAP^S127A^-transgenic mice compared to WT animals (8 animals/group). Statistical test: Mann-Whitney U. **p ≤ 0.01

To dissect how PAI-1 could contribute to YAP-driven tumorigenesis, expression profiling of Sk-Hep1 cells after PAI-1 inhibition was performed (scr. vs. siPAI-1). In total, 3174 mRNAs were significantly regulated after PAI-1 inhibition compared to control siRNA-transfected cells (at least +/− 1.3-fold change). While 1283 genes were upregulated (negatively regulated by PAI-1), 1891 transcripts were reduced (positively regulated by PAI-1). Interestingly, a *gene set enrichment analysis* (GSEA) revealed a significant enrichment of regulated genes in the process ‘cellular senescence’ (NES: − 1.77 and FDR = 0.002), (Fig. 5a/b). Indeed, the *senescence-associated secretory phenotype* (SASP) is characterized by the regulation of growth factors and chemokines as observed in our mouse model [14]. Accordingly, genes associated with SASP such as *insulin-like growth factor binding protein-3* (IGFBP3) or factors characteristic for replicative senescence (e.g. *checkpoint kinase-1*; CHK1) were repressed after PAI-1 silencing (Fig. 5c). Although the precise molecular mechanism of how PAI-1 controls transcriptional changes of these factors remains unclear, a potential role of PAI-1 in the regulation of senescence has already been discussed for other cell types [14, 15].

Because our data illustrated that YAP-regulated PAI-1 might affect cellular senescence, we investigated in the next step if a senescence-associated phenotype was detectable after YAP^S127A^ overexpression in vivo. In tumor nodules derived from YAP^S127A^ transgenic mice, elevated YAP and PAI-1 levels were observed (Fig. 5d). Additionally, positivity of the senescence marker ß-galactosidase was only measurable in YAP^S127A^-positive samples but not in WT liver tissues.

Together, these results strongly suggest that YAP-induced PAI-1 contributes to an oncogene-induced senescence phenotype.

### The YAP/TEAD4 protein complex regulates PAI-1 expression

Lastly, we aimed to understand the molecular mechanism how YAP controls PAI-1 expression. Because YAP is a transcriptional co-activator that does not contain a DNA binding site, it physically interacts with several *transcription factors* (TFs) to control target gene transcription. To define the precise mechanism how YAP regulates PAI-1, different known YAP-interacting TFs such as TEAD1, TEAD4 and FOXM1 were inhibited by RNAi and the effect on PAI-1 expression was analyzed [3, 16, 17].

**Fig. 6:**
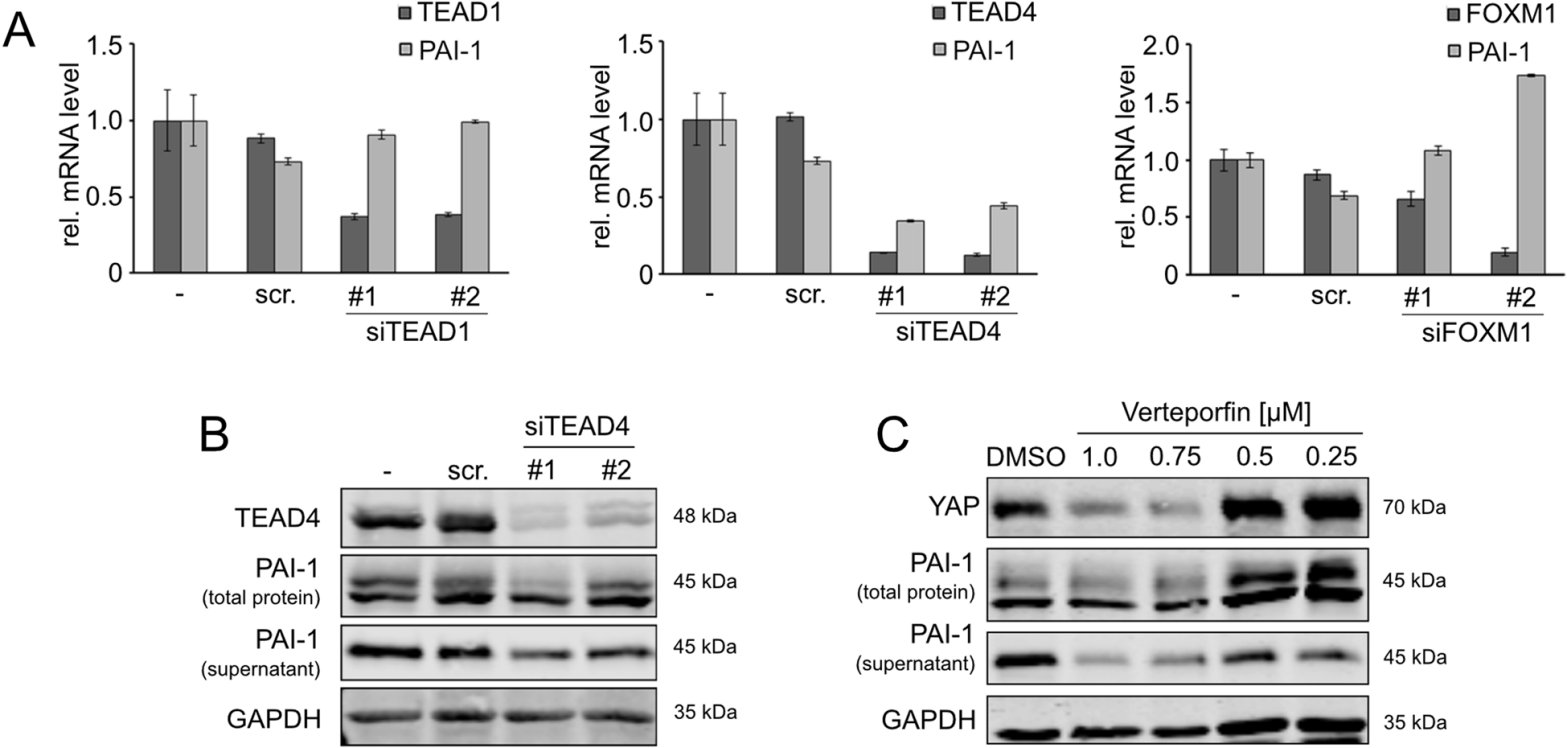
PAI-1 expression is regulated by the YAP/TEAD4 complex. **a** Real-time PCR analysis of PAI-1 mRNA levels after siRNA-mediated silencing of the known YAP-interacting transcription factors TEAD1, TEAD4 and FOXM1. Two independent siRNAs for each transcription factor were used (#1, #2) and compared to untreated (−) and scrambled siRNA-transfected SNU-182 cells (scr.). **b** Protein detection of secreted PAI-1 levels in the supernatant and total protein fractions derived from cultured Sk-Hep1 cells after siRNA mediated TEAD4-Inhibition. **c** Liver cancer cell line Sk-Hep1 was treated with increasing Verteporfin concentrations (0.25–1.0 μM) for 24 h followed by the detection of intracellular and secreted PAI-1. For **b** and **c**, signal intensity was measured and normalized to GAPDH. ‘scr’ controls represent samples that were transfected with the transfection reagent and non-specific siRNA (40 nM) with no similarity to cDNA

Silencing of TEAD4 but not of TEAD1 or FOXM1 was associated with reduced PAI-1 transcript levels (Fig. 6a). In addition, protein expression and secretion were diminished after TEAD4 inhibition (total PAI-1: down to 45%; secreted PAI-1: down to 43%, Fig. 6b). To further substantiate these results, Verteporfin was administered, which is known to disrupt the TEAD/YAP complex followed by degradation of YAP [18]. Indeed, YAP degradation was associated with reduced PAI-1 levels in cell lysates and cell culture supernatants after Verteporfin treatment in a concentration-dependent manner (total PAI-1: down to 79%; secreted PAI-1: down to 20%, Fig. 6c).

**Fig. 7:**
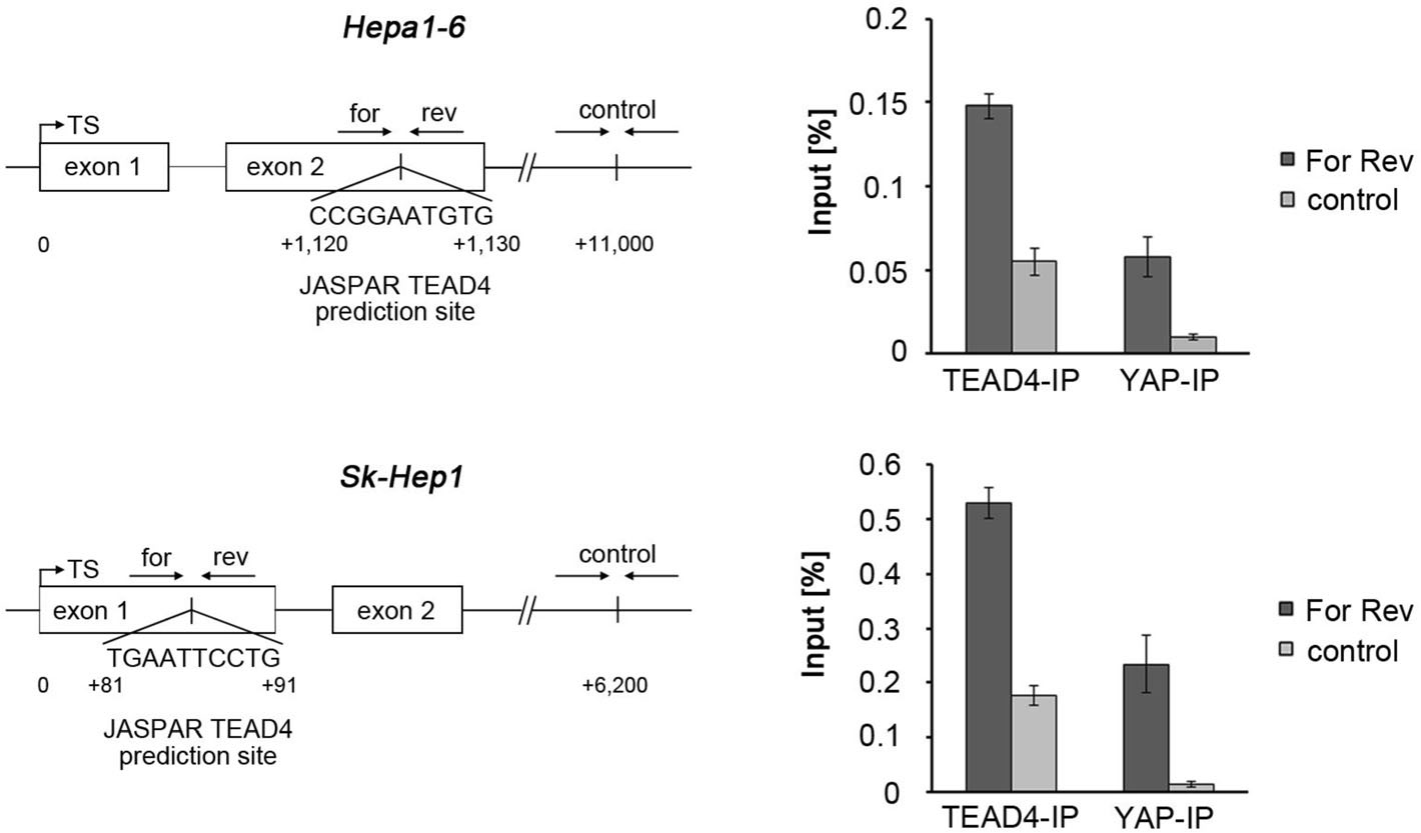
YAP and TEAD4 interact with the *SERPINE1* promoter. **a** ChIP analysis of TEAD4 and YAP binding to the murine (Hepa1–6) and human (Sk-Hep1) *SERPINE1* promoters at the indicated binding sites predicted by the JASPAR database. Schemes illustrate the potential binding sites and the downstream control site. TS – transcriptional start. Bars illustrate the relative binding capacity at the respective site in relation to the total input. **b** Luciferase assay after transient transfection of HEK-293 cells with pGL3-PAI-1 with and without siRNA-mediated inhibition of YAP or TEAD4 (2 siRNAs for each gene were tested). scr. - scrambled siRNA served as control. Coexpression of pRL-CMV-Renilla vector was used for normalization. **c** Coexpression of pGL3 (containing wildtype and mutated TEAD4 binding sites), pRL-CMV-Renilla (all samples, for normalization) and pDEST (with and without YAP) in HEK-293 cells

In the next step, we tested whether TEAD4 and YAP directly bind to the PAI-1 promoter of murine Hepa1–6 and human Sk-Hep1 cells. To identify potential TEAD4-binding sites, the JASPAR database was screened for promising sites in the PAI-1 gene (SERPINE1) and compared to publicly available TEAD4 ChIP-sequencing data sets of cancer cell lines [12, 19]. As a result, putative binding sites of TEAD4 were identified within the second exon of the murine PAI-1 gene and within the first exon of the human PAI-1 gene (see schemes Fig. 7a, left panel). *Chromatin immunoprecipitation* (ChIP) analysis illustrated binding of TEAD4 and YAP to the predicted binding sites in both analyzed cell lines, while significantly lower binding was observed for control regions downstream of the specific binding sites (Fig. 7a, right panel).

ChIP results were confirmed by a luciferase reporter assay showing that siRNA-mediated silencing of YAP or TEAD4 diminished reporter gene expression, which was under the control of the SERPINE1 promoter region containing the TEAD4 binding site (Fig. 7b). Equally, a point mutation in the TEAD4 binding site reduced luciferase expression about 45% compared to the wildtype site (Fig. 7c).

In summary, these results demonstrate the direct transcriptional regulation of PAI-1 by the YAP/TEAD4 complex as one mechanism responsible for increased PAI-1 secretion after YAP activation.

## Discussion

Based on transcriptomic and proteomic screening, this study illustrates that the oncogene YAP induces a secretory phenotype in liver tumorigenesis. Recent research demonstrates that an intense heterologous communication between liver resident cells such as hepatocytes, hepatic stellate cells, endothelial cells and Kupffer cells is required for tissue development, maintenance and regeneration [20]. Dysregulation of these multi-cellular communication networks can contribute to chronic liver diseases and tumorigenesis as illustrated for Notch1 signaling and its impact on tumor cell dissemination [21]. However, secreted factors may also affect neighboring cells already in premalignant conditions to reprogram their cellular functions or to create a tumor-supporting microenvironment [22]. To understand this heterologous secretome network and to identify promising points of interference for the development of therapeutic strategies, it is essential to decipher communication patterns connecting identical and different cell types in tumorigenesis.

For the Hippo signaling pathway, we here show that YAP regulates the secretion of chemokines and cytokines which are detectable in blood samples of YAP^S127A^ transgenic mice as illustrated for CXCL13, CXCL16 and PAI-1. On one hand, these factors represent potential communication hubs that connect YAP-overexpressing hepatocytes with other cells types such as infiltrating immune cells (e.g. CXCL13 stimulates IgG secretion by B cells or CXCL16-guided recruitment of NKT cells [23, 24];). On the other hand, secreted bioactive proteins may serve as biomarkers to identify groups of patients that are suitable for targeted therapy. In case of the Hippo pathway, specific gene cytokine/chemokine/growth factor signatures in the blood serum or plasma could characterize cancer patient groups that would benefit from a YAP-targeted therapy [1]. For example, elevated concentrations of proteins (e.g. PAI-1, CXCL13 and CXCL16) in the blood could indicate YAP activation in HCC tumor cells, which is detectable in about 30% of all HCC cases [3]. This risk-free and non-invasive method could guide clinicians to administer drugs and drug combinations in the future that include specific inhibitors for YAP [18, 25].

Indeed, approaches for the activation of the Hippo pathway and especially the inactivation of its negatively regulated downstream effector YAP are currently intensely investigated. First recently presented small compounds developed by different companies have been proposed as potential lead-compounds since they change YAP reporter assays, directly disturb physical interaction between YAP and TEADs [25], or change TEAD activity in a dominant-negative manner [26]. Our results indicate, that alternative treatment strategies could focus on communication networks induced by YAP in tumorigenesis. Here, perturbation of YAP-dependent intercellular communication (including PAI-1 as discussed below) could lead to a normalization of the tumor microenvironment, reduced tumor progression or even regression.

The protein PAI-1 drew our special attention, since it was not only clearly regulated by YAP in blood plasma samples in vivo but its expression was also significantly associated with poor clinical outcome. Like CTGF and CYR61, PAI-1 has already been described as regulated by YAP and Yorki (the YAP orthologue in *Drosophila melanogaster*) by high-throughput screening approaches [27], however, to our knowledge this is the first report demonstrating YAP/TEAD-dependent transcriptional regulation and secretion of PAI-1. PAI-1, which is encoded by the *SERPINE1* gene, acts as inhibitor of *tissue plasminogen activator* (tPA) and *urokinase plasminogen activator* (uPA) and has been described to correlate with poor patient prognosis of different tumor types including breast cancer [28, 29]. On the one hand, the prognostic relevance of PAI-1 overexpression may be related to its inhibitory effect on profibrinolytic plasmin associated with hypo-fibrinolytic conditions, which is commonly seen in patients with liver malignancies with venous thromboembolic complications [30]. On the other hand, PAI-1 expression has been described to modulate cancer cell proliferation and support tumorigenesis through its pro-angiogenic, pro-migratory and anti-apoptotic effects [15]. In addition, more recent data also demonstrates that PAI-1 controls cellular senescence and immune cell functionality as illustrated for macrophage migration and polarization [31–33].

Interestingly, no significant effect on cell viability, proliferation or migration were detectable after PAI-1 inhibition in our study (data not shown). It is therefore likely that further (YAP-induced) factors cooperating with PAI-1 are required to induce a prominent proliferative response. Instead, our data suggest that YAP-induced PAI-1 contributes to oncogene-induced senescence in vitro and in vivo. At first glance this seems contradicting since cellular senescence is considered to be a tumor-suppressive mechanism protecting cells from malignant transformation upon extra- or intracellular stress [34]. However, next to its safeguarding function, cellular senescence can also facilitate deleterious properties. This phenotype is characterized by SASP, consisting of actively secreted growth factors and chemokines [14]. These soluble signaling factors affect tumor and non-neoplastic cells to create a tumor-supportive microenvironment. The idea that YAP-dependent PAI-1 expression contributes to senescence is supported by previous data showing that in other experimental model systems PAI-1 is not only a bystander but also can stimulate replicative senescence [33]. Further studies are needed to decipher the specific role of PAI-1 in the context of SASP and its connection to YAP activation.

Due to its multiple tumor-promoting properties in several tumor entities, PAI-1 has also been considered as therapeutic target structure and several small compounds or antibodies specifically blocking PAI-1 activity have been developed [15, 35]. Some of these substances showed promising results in vitro and in vivo such as SK-116, which reduced serum PAI-1 levels and the number of intestinal polyps in mice with APC mutations [36]. However, no PAI-1-specific therapy has been tested in clinical trials, which is partly due to low stability of inhibitors and required high drug concentrations [15, 37]. Currently, it is questionable if therapies that exclusively target PAI-1 will be available, soon. Instead, combinatory silencing of PAI-1 together with other YAP-dependent secreted factors, may slow down tumor progression. One example for this kind of cooperative treatment is Maraviroc, which blocks the chemokine (C-C motif) receptor 5 (CCR5) that is activated by YAP-dependent CXCL13 [38]. It will be interesting to investigate if also the other identified secreted factors such as CXCL13 or CXCL16 are directly regulated by the YAP/TEAD4 complex or if additional YAP-interacting transcription factors such as FOXM1 or p73 contribute to the secretory phenotype observed in YAP-transgenic mice [3, 16, 39].

Although, the present study clearly illustrates that oncogenic YAP directly controls PAI-1 expression, the molecular impact on the other identified secreted factors such as CXCL13, CXCL16, PDGFB and PDGFC is less clear. Future studies not only must clarify if the YAP/TEAD complex directly controls transcription of the cytokines but also how consistent these effects are in different HCC cells or other tumor entities. This would be of special importance since the results will illustrate if PAI-1 and other cytokines could also serve as biomarkers or therapeutic target structures.

## Conclusions

This study illustrates that YAP-overexpressing hepatocytes and HCC cells control the expression of several paracrine-acting factors as exemplified for PAI-1. YAP is therefore responsible for a secretory phenotype that could connect tumorous and non-tumorous cell types in chronic liver disease and tumorigenesis. A comprehensive understanding of these communication networks will serve as basis to develop biomarkers and therapies targeting these points-of-interference with the aim to normalize heterologous communication patterns.

## Supplementary information

**Additional file 1.** Supplementary Tables S1-S4 and Supplementary Table S5).

## Data Availability

*Transcriptome data* generated and/or analyzed during the current study are available in the GEO database (GSE128043 for primary hepatocytes and GSE150796 for PAI-1 inhibition in Sk-Hep1 cells). The remaining data generated and analyzed during the current study are available from the corresponding author on reasonable request.

## Abbreviations

ChIP: Chromatin immunoprecipitation
CIN: Chromosome instability
CXCL13: C-X-C motif chemokine ligand 13
CXCL16: C-X-C motif chemokine ligand 16
CTGF: Connective tissue growth factor
CYR61: Cysteine-rich angiogenic inducer 61
ECM: Extracellular matrix
HCC: Hepatocellular carcinoma
PAI-1: Plasminogen activator inhibitor-1
RNAi: RNAinterference
SASP: Senescence-associated secretory phenotype
TEAD: TEA domain transcription factor
YAP: Yes-associated protein

## References

[1] Johnson R, Halder G (2014). The two faces of hippo: targeting the hippo pathway for regenerative medicine and cancer treatment. Nat Rev Drug Discov.

[2] Patel SH, Camargo FD, Yimlamai D (2017). Hippo signaling in the liver regulates organ size, cell fate, and carcinogenesis. Gastroenterology.

[3] Weiler SME, Pinna F, Wolf T, Lutz T, Geldiyev A, Sticht C, Knaub M, Thomann S, Bissinger M, Wan S (2017). Induction of chromosome instability by activation of yes-associated protein and Forkhead box M1 in liver Cancer. Gastroenterology.

[4] Lee KP, Lee JH, Kim TS, Kim TH, Park HD, Byun JS, Kim MC, Jeong WI, Calvisi DF, Kim JM, Lim DS (2010). The hippo-Salvador pathway restrains hepatic oval cell proliferation, liver size, and liver tumorigenesis. Proc Natl Acad Sci U S A.

[5] Song H, Mak KK, Topol L, Yun K, Hu J, Garrett L, Chen Y, Park O, Chang J, Simpson RM (2010). Mammalian Mst1 and Mst2 kinases play essential roles in organ size control and tumor suppression. Proc Natl Acad Sci U S A.

[6] Tschaharganeh DF, Chen X, Latzko P, Malz M, Gaida MM, Felix K, Ladu S, Singer S, Pinna F, Gretz N (2013). Yes-associated protein up-regulates Jagged-1 and activates the notch pathway in human hepatocellular carcinoma. Gastroenterology.

[7] Weiler SME, Lutz T, Bissinger M, Sticht C, Knaub M, Gretz N, Schirmacher P, Breuhahn K (2020). TAZ target gene ITGAV regulates invasion and feeds back positively on YAP and TAZ in liver cancer cells. Cancer Lett.

[8] Yabuta N, Mukai S, Okamoto A, Okuzaki D, Suzuki H, Torigata K, Yoshida K, Okada N, Miura D, Ito A (2013). N-terminal truncation of Lats1 causes abnormal cell growth control and chromosomal instability. J Cell Sci.

[9] Zhou TY, Zhou YL, Qian MJ, Fang YZ, Ye S, Xin WX, Yang XC, Wu HH (2018). Interleukin-6 induced by YAP in hepatocellular carcinoma cells recruits tumor-associated macrophages. J Pharmacol Sci.

[10] Camargo FD, Gokhale S, Johnnidis JB, Fu D, Bell GW, Jaenisch R, Brummelkamp TR (2007). YAP1 increases organ size and expands undifferentiated progenitor cells. Curr Biol.

[11] Vandesompele J, De Preter K, Pattyn F, Poppe B, Van Roy N, De Paepe A, Speleman F (2002). Accurate normalization of real-time quantitative RT-PCR data by geometric averaging of multiple internal control genes. Genome Biol.

[12] Mathelier A, Zhao X, Zhang AW, Parcy F, Worsley-Hunt R, Arenillas DJ, Buchman S, Chen CY, Chou A, Ienasescu H (2014). JASPAR 2014: an extensively expanded and updated open-access database of transcription factor binding profiles. Nucleic Acids Res.

[13] Roessler S, Jia HL, Budhu A, Forgues M, Ye QH, Lee JS, Thorgeirsson SS, Sun Z, Tang ZY, Qin LX, Wang XW (2010). A unique metastasis gene signature enables prediction of tumor relapse in early-stage hepatocellular carcinoma patients. Cancer Res.

[14] Coppe JP, Desprez PY, Krtolica A, Campisi J (2010). The senescence-associated secretory phenotype: the dark side of tumor suppression. Annu Rev Pathol.

[15] Kubala MH, DeClerck YA (2019). The plasminogen activator inhibitor-1 paradox in cancer: a mechanistic understanding. Cancer Metastasis Rev.

[16] Eisinger-Mathason TS, Mucaj V, Biju KM, Nakazawa MS, Gohil M, Cash TP, Yoon SS, Skuli N, Park KM, Gerecht S, Simon MC (2015). Deregulation of the hippo pathway in soft-tissue sarcoma promotes FOXM1 expression and tumorigenesis. Proc Natl Acad Sci U S A.

[17] Zhao B, Ye X, Yu J, Li L, Li W, Li S, Yu J, Lin JD, Wang CY, Chinnaiyan AM (2008). TEAD mediates YAP-dependent gene induction and growth control. Genes Dev.

[18] Liu-Chittenden Y, Huang B, Shim JS, Chen Q, Lee SJ, Anders RA, Liu JO, Pan D (2012). Genetic and pharmacological disruption of the TEAD-YAP complex suppresses the oncogenic activity of YAP. Genes Dev.

[19] Encode Project Consortium (2012). An integrated encyclopedia of DNA elements in the human genome. Nature.

[20] Camp JG, Sekine K, Gerber T, Loeffler-Wirth H, Binder H, Gac M, Kanton S, Kageyama J, Damm G, Seehofer D (2017). Multilineage communication regulates human liver bud development from pluripotency. Nature.

[21] Wieland E, Rodriguez-Vita J, Liebler SS, Mogler C, Moll I, Herberich SE, Espinet E, Herpel E, Menuchin A, Chang-Claude J (2017). Endothelial Notch1 activity facilitates metastasis. Cancer Cell.

[22] Nussbaum T, Samarin J, Ehemann V, Bissinger M, Ryschich E, Khamidjanov A, Yu X, Gretz N, Schirmacher P, Breuhahn K (2008). Autocrine insulin-like growth factor-II stimulation of tumor cell migration is a progression step in human hepatocarcinogenesis. Hepatology.

[23] Geissmann F, Cameron TO, Sidobre S, Manlongat N, Kronenberg M, Briskin MJ, Dustin ML, Littman DR (2005). Intravascular immune surveillance by CXCR6+ NKT cells patrolling liver sinusoids. PLoS Biol.

[24] Li C, Kang D, Sun X, Liu Y, Wang J, Gao P (2015). The effect of C-X-C motif chemokine 13 on hepatocellular carcinoma associates with Wnt signaling. Biomed Res Int.

[25] Calses PC, Crawford JJ, Lill JR, Dey A (2019). Hippo pathway in Cancer: aberrant regulation and therapeutic opportunities. Trends Cancer.

[26] Holden JK, Crawford JJ, Noland CL, Schmidt S, Zbieg JR, Lacap JA, Zang R, Miller GM, Zhang Y, Beroza P (2020). Small molecule Dysregulation of TEAD Lipidation induces a dominant-negative inhibition of hippo pathway signaling. Cell Rep.

[27] Liu F, Lagares D, Choi KM, Stopfer L, Marinkovic A, Vrbanac V, Probst CK, Hiemer SE, Sisson TH, Horowitz JC (2015). Mechanosignaling through YAP and TAZ drives fibroblast activation and fibrosis. Am J Physiol Lung Cell Mol Physiol.

[28] Li S, Wei X, He J, Tian X, Yuan S, Sun L (2018). Plasminogen activator inhibitor-1 in cancer research. Biomed Pharmacother.

[29] Palmirotta R, Ferroni P, Savonarola A, Martini F, Ciatti F, Laudisi A, Sini V, Del Monte G, Guadagni F, Roselli M (2009). Prognostic value of pre-surgical plasma PAI-1 (plasminogen activator inhibitor-1) levels in breast cancer. Thromb Res.

[30] Carmeliet P, Stassen JM, Schoonjans L, Ream B, van den Oord JJ, De Mol M, Mulligan RC, Collen D (1993). Plasminogen activator inhibitor-1 gene-deficient mice. II. Effects on hemostasis, thrombosis, and thrombolysis. J Clin Invest.

[31] Ichimura A, Matsumoto S, Suzuki S, Dan T, Yamaki S, Sato Y, Kiyomoto H, Ishii N, Okada K, Matsuo O (2013). A small molecule inhibitor to plasminogen activator inhibitor 1 inhibits macrophage migration. Arterioscler Thromb Vasc Biol.

[32] Kubala MH, Punj V, Placencio-Hickok VR, Fang H, Fernandez GE, Sposto R, DeClerck YA (2018). Plasminogen activator Inhibitor-1 promotes the recruitment and polarization of macrophages in Cancer. Cell Rep.

[33] Kortlever RM, Higgins PJ, Bernards R (2006). Plasminogen activator inhibitor-1 is a critical downstream target of p53 in the induction of replicative senescence. Nat Cell Biol.

[34] Campisi J (2007). d'Adda di Fagagna F: cellular senescence: when bad things happen to good cells. Nat Rev Mol Cell Biol.

[35] Rouch A, Vanucci-Bacque C, Bedos-Belval F, Baltas M (2015). Small molecules inhibitors of plasminogen activator inhibitor-1 - an overview. Eur J Med Chem.

[36] Mutoh M, Niho N, Komiya M, Takahashi M, Ohtsubo R, Nakatogawa K, Ueda K, Sugimura T, Wakabayashi K (2008). Plasminogen activator inhibitor-1 (Pai-1) blockers suppress intestinal polyp formation in min mice. Carcinogenesis.

[37] Gorlatova NV, Cale JM, Elokdah H, Li D, Fan K, Warnock M, Crandall DL, Lawrence DA (2007). Mechanism of inactivation of plasminogen activator inhibitor-1 by a small molecule inhibitor. J Biol Chem.

[38] Pervaiz A, Zepp M, Mahmood S, Ali DM, Berger MR, Adwan H (2019). CCR5 blockage by maraviroc: a potential therapeutic option for metastatic breast cancer. Cell Oncol (Dordr).

[39] Strano S, Monti O, Pediconi N, Baccarini A, Fontemaggi G, Lapi E, Mantovani F, Damalas A, Citro G, Sacchi A (2005). The transcriptional coactivator yes-associated protein drives p73 gene-target specificity in response to DNA damage. Mol Cell.

