# Supplementary material for: Yes-associated protein (YAP) induces a secretome phenotype and transcriptionally regulates plasminogen activator Inhibitor-1 (PAI-1) expression in hepatocarcinogenesis
Source: Cell Commun Signal. 2020 Oct 23;18:166. doi: 10.1186/s12964-020-00634-6 (PMC7583285; doi:10.1186/s12964-020-00634-6)
Supplement: Supplementary file 2 — Additional file 1. Supplementary Tables S1-S4 and Supplementary Table S5). [file 12964_2020_634_MOESM2_ESM.zip › Supplementary Tables S1-S4_CCAS-D-20-00140.R1.docx]

**Supplementary Material**

*Supplementary Table S1: siRNA sequences*

| **siRNA** | **mRNA Accession No.** | **Sequence (5'→3')** |
| --- | --- | --- |
| *hFOXM1 #1* | NM_202003 | AUAUUCACAGCAUCAUCAC-dT-dT |
| *hFOXM2 #2* |  | GGACCACUUUCCCUACUUU-dT-dT |
| *hPAI-1 #1* | NM_000602.4 | AAGTGAAGATCGAGGTGAA-dT-dT |
| *hPAI-1 #2* |  | AAGGATGAGATCAGCACCACA-dT-dT |
| *hTEAD1 #1* | NM_021961.5 | GGACAUUCGUCAGAUUUAUGA-dT-dT |
| *hTEAD1 #2* |  | GAGCACAACAUCAUAUUUACA-dT-dT |
| *hTEAD4 #1* | NM_201443.2 | AGGUACUUCCUCAAUCACA-dT-dT |
| *hTEAD4 #2* |  | CCGCCAAAUCUAUGACAAA-dT-dT |
| *hYAP #1* | NM_006106 | CCACCAAGCUAGAUAAAGA-dT-dT |
| *hYAP #2* |  | GGUCAGAGAUACUUCUUAA-dT-dT |
| *mYAP #1* | NM_001171147 | CCACCAAGCUAGAUAAAGA-dT-dT |
| *mYAP #2* |  | GGAGAAGUUUACUACAUAA-dT-dT |
| *Scrambled* | - | UGGUUUACAUGUCGACUAA-dT-dT |

h - human; m - murine

*Supplementary Table S2: primer sequences for ChIP analysis*

| **Gene** | **Sequence (5’→3’)** |
| --- | --- |
| *hSERPINE1 promoter* | For: GCACAGCTGTGTTTGGCTGC  Rev: CTACCTGAAGTTCTCAGAGGTGCC |
| *hSERPINE1 downstream control* | For: GCGACAGAGTGAGACTCCTTCTC  Rev: GTGTGTAAGAGACAGGGTCTCACTG |
| *mSerpine1 promoter* | For: GAGAATCCCACACAGCCCATCAG  Rev: CGCTGTGGTCATCTGCAGCATAG |
| *mSerpine1 downstream control* | For: GGCAGGCAAGATGGCTCAGATG  Rev: GTATGCAAGTCAGAGGACAACCC |

h - human; m - murine

*Supplementary Table S3: Antibodies*

| **Antigene (clone)** | **Dilution** | **Source** |
| --- | --- | --- |
| β-Actin (C4) | WB: 1:10000 | MP Biomedicals, Santa Ana (USA) |
| Albumin | WB: 1:1000 | Cell Signaling, Frankfurt (Germany) |
| GAPDH | WB: 1:10000 | Millipore, Darmstadt (Germany) |
| GAPDH (A-3) | WB: 1:300 | Santa Cruz Biotechnology, Heidelberg (Germany) |
| PAI-1 (rabbit) | WB: 1:1000  IHC: 1:50 | Thermo Fisher Scientific, Darmstadt (Germany) |
| PAI-1 (mouse) | WB: 1:500 | Thermo Fisher Scientific (Germany) |
| TEAD4 | WB: 1:500 | Abcam, Cambridge (UK) |
| TEF-3 (N-G2) | ChIP: 2 µg | Santa Cruz Biotechnology (USA) |
| β-Tubulin (TUB 2.1) | WB: 1:500 | Santa Cruz Biotechnology (USA) |
| YAP | WB: 1:400 | Cell Signaling (Germany) |
| YAP (D8H1X) | ChIP: 1:50  IHC: 1:200 | Cell Signaling (Germany) |

*Supplementary Table S4: Primer sequences for real-time PCR*

| **Gene** | **mRNA accession No.** | **Sequence (5’→3’)** |
| --- | --- | --- |
| *hB2M* | NM_004048 | For: CACGTCATCCAGCAGAGAAT  Rev: TGCTGCTTACATGTCTCGAT |
| *hCHEK1* | NM_001114122.2 | For: GAAGACTGGGACTTGGTGC  Rev: CCTTCTCTCCTGTGACCATAG |
| *hFOXM1* | NM_202003 | For: ATAGCAAGCGAGTCCGCATT  Rev: TTCCTCCCCAGGCTGGATTT |
| *hIGFBP3* | NM_001013398.2 | For: GCTCTGCGTCAACGCTAGTG  Rev: GCATGCCCTTTCTTGATGATG |
| *hRPL41* | NM_001035267 | For: AAACCTCTGCGCCATGAGAG  Rev: AGCGTCTGGCATTCCATGTT |
| *hSERPINE1* | NM_000602.4 | For: GCAGCAGATTCAAGCAGCTATG  Rev: CTTCAGATCCCGCTGGACGAAG |
| *hTEAD1* | NM_021961.5 | For: GACAGTCACCTGTTCCACCAAAG  Rev: CCATTCTCAAACCTTGCATACTCCG |
| *hTEAD4* | NM_201443.2 | For: TGGAGTTCTCTGCCTTCCTG  Rev: GGACTGGCCAATGTGCACGA |
| *hYAP1* | NM_006106 | For: CCTGCGTAGCCAGTTACCAA  Rev: CCATCTCATCCACACTGTTC |
| *mActb* | NM_007393 | For: GCTTCTTTGCAGCTCCTTCGT  Rev: ACCAGCGCAGCGATATCG |
| *mAlbumin* | BC_049971.1 | For: GAGGCTGCAAGAAACCTAGGAAGAG  Rev: CACCAGGGATCCACTACAGCAC |
| *mCtgf* | NM_010217.2 | For: GGAGAACTGTGTACGGAGCG  Rev: CCAGGCAAGTGCATTGGTA |
| *mCcl5* | NM_013653 | For: CACCATCATCCTCACTGCAGC  Rev: GCACTTGCTGCTGGTGTAGAAATAC |
| *mCxcl10* | NM_021274 | For: GGTCCGCTGCAACTGCATCC  Rev: GATTCCGGATTCAGACATCTCTGC |
| *mCxcl13* | BC_012965 | For: GCAACGCTGCTTCTCCTCCTG  Rev: GGCGTAACTTGAATCCGATCTATG |
| *mCxcl16* | NM_023158 | For: GCTTTGGACCCTTGTCTCTTGC  Rev: GGGTGCCAGAAGAAATGGTACGATC |
| *mCyr61* | NM_010516 | For: GATCTGTGAAGTGCGTCCTTGTGG  Rev: GACACTGGAGCATCCTGCATAAG |
| *mGapdh* | NM_008084 | For: TGTCCGTCGTGGATCTGAC  Rev: CCTGCTTCACCACCTTCTTG |
| *mGdf15* | NM_011819.3 | For: GAGGACTCGAACTCAGAACCAAGTC  Rev: GAGTAGCAGCTGGCCGTG |
| *mHprt* | NM_013556 | For: TCCTCCTCAGACCGCTTTT  Rev: CCTGGTTCATCATCGCTAATC |
| *mPdgfa* | NM_008808.3 | For: CTGTTGTAACACCAGCAGCGTC  Rev: GGCTTCTTCCTGACATACTCCAC |
| *mPdgfb* | NM_011057.3 | For: CCTCGGCCTGTGACTAGAAGTC  Rev: GAATGGTCACCCGAGCTTGAG |
| *mPdgfc* | AF_286725 | For: GTAGAAGTTGAGGAGCCCAGTGATG  Rev: GTCACAGCATTGTTGAGCAGGTC |
| *mPpia* | NM_008907 | For: GCATACAGGTCCTGGCATCT  Rev: AGCTGTCCACAGTCGGAAAT |
| *mSerpine1* | NM_008871.2 | For: GACTTCTCAGAAGTGGAAAGAGCC  Rev: CTGAAGTAGAGGGCATTCACCAGC |
| *mVegfa* | NM_001287056 | For: CCAAAGAAAGACAGAACAAAGCCAG  Rev: CGCTCCAGGATTTAAACCGG |
| *mVegfb* | BC_046303 | For: GTGTGACTGTGCAGCGCTGTG  Rev: GCATTCACATTGGCTGTGTTCTTC |
| *mVegfc* | NM_009506 | For: CAGCGTAGATGAGCTGATGTCTGTC  Rev: GAAGGTGTTTGTGGCTGCTCC |
| *mYap* | NM_001171147 | For: GACTCCGAATGCAGTGTCTT  Rev: ATCGGAACTATTGGTTGTCA |

h - human; m - murine
